# Oral health experiences of individuals with Rett syndrome: a retrospective study

**DOI:** 10.1186/s12903-018-0651-y

**Published:** 2018-11-29

**Authors:** Y.Y.L. Lai, K. Wong, N. M. King, J. Downs, H. Leonard

**Affiliations:** 1 Department of Paediatric Dentistry, School of Dentistry, The University of Western Australia, Perth, WA Australia; 2 Telethon Kids Institute, The University of Western Australia, Perth, WA Australia; 3 School of Physiotherapy and Exercise Science, Curtin University, Perth, Australia

**Keywords:** Rett syndrome, Dental manifestation, Oral health experience, Rare disorder, Developmental disability, MECP2

## Abstract

**Background:**

There is relatively little literature on the oral health experiences of individuals with Rett syndrome. This study described the incidence of dental extractions and restorations in a population-based cohort, according to a range of demographic and clinical factors. The association between bruxism and age was also investigated.

**Methods:**

Existing questionnaire data in the population-based Australian Rett Syndrome Database for the years 2004, 2006, 2009 or 2011 on genetically confirmed female cases (*n* = 242) were analysed.

**Results:**

The incidence rate of restorations and extractions were 6.8 per 100 person years (py) and 9.3 per 100 py respectively. The incidence of extractions decreased with increasing levels of income. Compared to those with a C-terminal mutation, the incidence rate of extraction was higher for those with large deletions (Incidence Rate Ratio (IRR) 4.93; 95% CI 1.46–16.7, *p* = 0.01). There was a 5% decrease in the risk of frequent bruxism for every one-year increase in age (Risk Ratio 0.95; 95% CI 0.94–0.97).

**Conclusions:**

Social advantage may provide some protection for dental health in individuals with Rett syndrome. Those with more severe genotypes seemed to have poorer oral health outcomes.

## Introduction

Rett syndrome (RTT) is a neurodevelopmental disorder affecting mostly females [1, 2] and occurring in 1 in 9000 females [3]. Although reported initially by Andreas Rett in 1966 [4], the condition did not become known in the English literature until Hagberg published a report of 35 cases in 1983 [5]. Cardinal features are the occurrence of regression after a period of relative normalcy [6], severe intellectual disability [1], stereotypic hand movements [7–9] as well as a characteristic teeth-grinding [10]. Other features include altered breathing patterns such as hyperventilation and breath holding [11–13], poor sleep [14–16], the development of seizures [10], scoliosis [17], feeding and gastrointestinal problems [18–21], failure to thrive [22–24], small feet and hands [25], and the development of osteoporosis [26].

Caused by a mutation in the the methyl-CpG-binding protein 2 (*MECP2*) gene [27–30], evidence suggests that much of the clinical variation evident in RTT is related to genotype [31]. Generally, p.Arg133Cys and p.Arg294* mutations are considered the mildest mutations, with p.Arg270*, p.Arg255* and large deletions the most severe, with significant differences in characteristics such as ambulation, hand use and language [31, 32].

There is little published dental literature about individuals with RTT although bruxism is a commonly described feature [1, 33–39]. In one early study comparing the behavioural phenotype of females with RTT (*n* = 143) and those with severe mental retardation (SMR) (*n* = 85), teeth grinding was reported in 86.0% of those with RTT and 44.7% in the SMR group (*p* < 0.001) [40]. Another study concluded that diurnal bruxism appeared to be indicative of the presence of a *MECP2* mutation in a child with clinical features of RTT [41]. Dental findings reported have included an anterior open-bite as well as palatal shelving possibly in relation to mouth breathing, digit sucking and mouthing [1, 34, 35]. Gingivitis [1, 36] and periodontal changes [35] have also been reported as has non-physiologic tooth wear [1, 34, 35, 38]. Gastroesophageal reflux has been frequently described in those with RTT [19] and may contribute to dental erosion, however, to date, no studies have specifically attributed non-physiological tooth wear to erosion due to gastroesophageal reflux.

The experience of dental care in individuals with RTT has only been reported once to date in a Spanish study involving 41 females with RTT and 82 age-matched control subjects [35]. Given the paucity of information on the dental problems and experience of accessing dental treatment for individuals with RTT, this study sought evidence, in an Australian population of girls and women with RTT, of 1) the incidence of dental extractions and restorations; 2) the prevalence of dental visits and treatment items; 3) the association of the latter two items with geographical location, income, mutation, presence of epilepsy, and presence of gastric reflux and 4) the relationship of dental bruxism with age.

## Methods

### Data source

Established in 1993, the Australian Rett Syndrome Database (ARSD) is an ongoing population-based registry of females with RTT born since 1976 [42]. Longitudinal data have been collected through questionnaires administered to caregivers approximately every two to four years since 2000 [17]. These cover a range of topics on child and family health and wellbeing. A section on oral health was first included in 2004 and these questions have been modified further in subsequent questionnaires in 2006, 2009 and 2011.

For inclusion in this study the subjects had to have a confirmed genetic diagnosis and the family also had to have submitted at least one follow-up questionnaire in either 2004, 2006, 2009 or 2011.

### Variables

#### Family factors

The Accessibility/Remoteness Index of Australia Plus (ARIA+) is an index used to quantify geographical location according to the road distance to major service centres in Australia. The ARIA+ score is categorised into major cities of Australia, inner regional Australia, outer regional Australia, remote Australia or very remote Australia [43]. Using all available data points, each individual was assigned the ARIA+ score that corresponded to their residential location.

Family income was used as a measure of socioeconomic status. The family income bracket assigned to each case was based on income from the latest returned questionnaire. Income brackets were classified overall as “less than $31 200”, “between $31 200 - $51 999”, “between $52 000 - $77 999” and “$78 000 or more” to accommodate changes in the income brackets between 2004 and 2011 .

#### Child factors

Age groups were defined to best approximate the primary dentition (0 to 6 years), mixed dentition (6 to 12 years), and permanent dentition (12 to 19 years; 19 years and above) phases of dental development. The time under observation for extractions and restorations respectively varied according to the nature of the follow-up questionnaire, whether the questionnaire was a first or subsequent follow-up questionnaire and whether it was completed by a parent or other caregiver. Therefore, the working age used in the incidence calculations for restorations and extractions was the midpoint of age during the time period to which the follow up questionnaire referred.

*MECP2* mutation types were categorised as C-terminal deletions, p.Arg106Trp, p.Arg133Cys, p.Arg168*, p.Arg255*, p.Arg270*, p.Arg294*, p.Arg306Cys, p.Thr158Met, large deletions and early truncating deletions [32]. All other *MECP2* mutations were categorised as “other”.

Mobility was classified according to whether the girl or woman had learned to walk (“independent”), walked with assistance (“assisted”), never learned to walk (“wheelchair”) or deteriorated from independent/assisted walking to being unable to walk (“deteriorating walking status”) according to a previously published latent class model [17]. Epilepsy and gastric reflux were captured using binary variables indicating whether or not ever diagnosed. Bruxism was classified as “0- none”, “1- slight” or “2- often” and taken directly from the bruxism item from the Rett Syndrome Behaviour Scale [44].

#### Oral health experiences

Information on dental attendance and previous dental treatment was restricted to data collected in the 2009 and 2011 family questionnaires which asked whether the individual had attended a dental visit or received any dental treatment under general anaesthesia (GA) during the calendar year in which the questionnaire was administered. Data from a subsequent section of the same questionnaire on hospital admissions as well as hospital records were used to validate or supplement this data [45].

#### Incidence calculations

The incidence rates of dental extractions (number of teeth extracted) and restorations were calculated by the number of new events during a period divided by the person-time at risk of experiencing the event. If a full dental clearance was performed (*n* = 3), the estimated number of teeth removed according to dental age and accounting for previous extractions was used. On the other hand, if the response indicated extractions but no number was given (*n* = 12), the mode (mode = 1) was used in the analysis.

### Data analysis

Data were analysed using STATA/IC Version 13.0. Incidence rates (using the exact Poisson method for estimation of confidence interval) of data extractions and restorations were reported by age group, ARIA+ group, income group, mutation group, mobility status, and history of epilepsy and reflux. Negative binomial regression was used to estimate the incidence rate ratio according to these factors. For longitudinal data, generalised linear models were used to estimate the incidence rate ratio of data extractions and restorations and likelihood of frequent bruxism by age, including the use of generalised estimating equations to adjust for correlated data. For bruxism, the “margins” command in Stata was used to determine predictive risk of bruxism by age. Proportions of attendance at dental visits, and receipt of dental treatment under GA were compared by geographical remoteness, income, mutation, presence of epilepsy, level of mobility, and presence of gastric reflux using the Fisher’s exact test of independence. All data analyses were carried out using Stata/IC version 13.0.

Ethics approval for this study was received from the Human Research Ethics Committee of Princess Margaret Hospital (Approval No. 1909/EP).

## Results

There were 242 eligible cases, 44 of whom were deceased at the time of the study. Descriptive characteristics of the study population are shown in Table 1. Of the 242 cases, 61.6% lived in metropolitan areas, 23.1% in inner regional areas, 10.3% in outer regional areas and 4.6% lived in remote or very remote areas of Australia. Fifty-three (21.9%) of the families reported earning less than $31,200 per annum (p.a.), with a similar proportion (21.5%) earning $78,000 p.a. or more. Each of the common *MECP2* mutation groups were represented. More than one third (39.7%) of individuals were independently mobile, with 14.5% having assisted mobility, 32.6% being wheelchair dependent, and in 12.4%, mobility had deteriorated. The majority of the cohort (81.8%) had been diagnosed with epilepsy, while just over a quarter (26.4%) had previous or current symptoms of gastric reflux.

**Table 1:** Population descriptive data^a^

Covariate	Covariate subcategory	*n* (%)
ARIA+	1 (major cities of Australia)	149 (61.6)
	2 (inner regional Australia)	56 (23.1)
	3 (outer regional Australia)	25 (10.3)
	4 (remote Australia/very remote Australia)	11 (4.6)
	Missing	1 (0.4)
Income	Less than $31,200	53 (21.9)
	Between $31,200 - $51,999	32 (13.2)
	Between $52,000 - $77,999	26 (10.7)
	$78,000 or more	52 (21.5)
	Missing	79 (32.6)
Mutation	C-terminal	23 (9.5)
	Early truncating	16 (6.6)
	Large deletion	17 (7.0)
	p.Arg106Trp	11 (4.6)
	p.Arg133Cys	20 (8.3)
	p.Arg168^*^	28 (11.6)
	p.Arg255^*^	17 (7.0)
	p.Arg270^*^	19 (7.9)
	p.Arg294^*^	20 (8.3)
	p.Arg306Cys	14 (5.8)
	p.Thr158Met	26 (10.7)
	Other	26 (10.7)
	Missing	5 (2.1)
Mobility	Independent	96 (39.7)
	Assisted	35 (14.5)
	Wheelchair	79 (32.6)
	Deteriorate	30 (12.4)
	Missing	2 (0.8)
Epilepsy	No	43 (17.8)
	Yes	198 (81.8)
	Missing	1 (0.4)
Reflux	No	178 (73.6)
	Yes	64 (26.4)

^a^*n* = 242

### Incidence of restorations

The incidence rates of restorations by age group and covariates are presented in Table 2 and the incidence rate ratios in Table 3. The total incidence of restorations was 6.8 restorations per 100 person-years (py) equating to almost seven restorations if ten individuals were followed for ten years. By using the midpoint of age during the time period to which follow-up referred and excluding first time point, the incidence was 13.8 per 100 py for those aged 6 years or less, 11.7 per 100 py for those 7–12 years old, 19.1 per 100 py for those 13–19 years, and 12.4 per 100 py for those 20 years and over (Table 2).

**Table 2:** Incidence of extractions and restorations by co-variates^c^

				Restorations			Extractions^b^		
Covariate	Covariate subcategory	*n* (%)	Time period (yrs)	Number	Incidence (per 100 py)	95% CI^a^	Number	Incidence (per 100 py)	95% CI^a^
Age group^d^	6 yrs and less	40^+^	130.16	18	13.8	n/a	13	10.0	n/a
	7–12 yrs	75^+^	324.14	38	11.7	n/a	41	12.6	n/a
	13–19 yrs	83^+^	360.73	69	19.1	n/a	43	11.9	n/a
	20 yrs and over	74^+^	436.53	54	12.4	n/a	104	23.8	n/a
	Total (age group calculations)	272^+^	1251.56	179	14.3	n/a	201	16.1	n/a
ARIA+	1 (major cities of Australia)	146 (61.6)	2447.3	151	6.2	5.2, 7.2	218	8.9	7.8, 10.2
*n* = 237	2 (inner regional Australia)	55 (23.2)	1084.6	70	6.5	5.0, 8.2	126	11.6	9.7, 13.8
	3 (outer regional Australia)	25 (10.6)	421.0	39	9.3	6.6, 12.7	36	8.6	6.0, 11.8
	4 (remote/very remote Australia)	11 (4.6)	194.8	22	11.3	7.1, 17.1	8	4.1	1.8, 8.1
Income	Less than $31,200	53 (32.5)	913.8	71	7.8	6.1, 9.8	120	13.1	10.9, 15.7
*n* = 162	Between $31,200 - $51,999	32 (19.6)	548.9	43	7.8	5.7, 10.6	36	6.6	4.6, 9.1
	Between $52,000 - $77,999	26 (15.9)	390.8	33	8.4	5.8, 11.9	16	4.1	2.4, 6.6
	$78,000 or more	52 (31.9)	750.4	56	7.5	5.6, 9.7	45	6.0	4.4, 8.0
Mutation	C-terminal	22 (9.4)	421.4	33	7.8	5.4, 11.0	20	4.7	2.9, 7.3
*n* = 234	Early truncating	16 (6.8)	248.9	15	6.0	3.4, 9.9	19	7.6	4.6, 11.9
	Large deletion	17 (7.3)	245.8	16	6.5	3.7, 10.6	50	20.3	15.1, 26.8
	p.Arg106Trp	11 (4.7)	181.8	3	1.7	0.3, 4.8	24	13.8	8.9, 20.3
	p.Arg133Cys	20 (8.6)	333.4	22	6.6	4.1, 10.0	43	12.9	9.3, 17.4
	p.Arg168*	28 (12.0)	466.8	51	10.9	8.1, 14.4	36	7.7	5.4, 10.7
	p.Arg255*	17 (7.3)	273.1	6	2.2	0.8, 4.8	21	7.7	4.8, 11.8
	p.Arg270*	18 (7.7)	358.8	19	5.3	3.2, 8.3	50	13.9	10.3, 18.4
	p.Arg294*	20 (8.6)	389.7	19	4.9	2.9, 7.6	31	8.0	5.4, 11.3
	p.Arg306Cys	14 (6.0)	280.0	23	8.2	5.2, 12.3	15	5.4	3.0, 8.8
	p.Thr158Met	25 (10.7)	405.5	24	5.9	3.8, 8.8	31	8.1	5.6, 11.4
	Other	26 (11.1)	501.0	43	8.6	6.2, 11.6	37	7.4	5.2, 10.2
Epilepsy	No	42 (17.6)	571.58	30	5.2	3.5, 7.5	67	11.7	9.1, 14.9
n = 237	Yes	196 (82.4)	3591.96	252	7.0	6.2, 7.9	321	8.9	8.0, 10.0
Reflux	No	174 (73.1)	3024.03	210	6.9	6.0, 7.9	278	9.2	8.1, 10.3
*n* = 238	Yes	64 (26.9)	1139.51	72	6.3	4.9, 8.0	110	9.7	7.9, 11.6

^a^Confidence interval was calculated using the exact Poisson method ^b^refers to number of extracted teeth ^c^*n* = 238

^d^Using all except questionnaire data from first time points, and using median age for all time periods ^+^number of time points included in analysis

**Table 3:** Incidence rate ratios of extractions and restorations by range of co-variates

			Baseline comparison	
Covariate	Covariate Subcategory	*n* (%)	Restorations	Extractions^a^
			Incidence rate ratio (IRR; (95% CI) *p*-value)	Incidence rate ratio (IRR; (95% CI) p-value)
ARIA+	1 (major cities of Australia)	146 (61.6)	baseline comparison	
n = 237	2 (inner regional Australia)	55 (23.2)	(1.40; (0.66–2.97) *p* = 0.38)	(1.10; (0.61–2.00) *p* = 0.74)
	3 (outer regional Australia)	25 (10.6)	(1.64; (0.59–4.52) *p* = 0.34)	(1.09; (0.47–2.53) *p* = 0.84)
	4 (remote Australia/very remote Australia)	11 (4.6)	(1.86; (0.44–7.82) *p* = 0.39)	(0.35; (0.1–1.30) *p* = 0.12)
Income	Less than $31,200	53 (32.5)	baseline comparison	
n = 162	Between $31,200 - $51,999	32 (19.6)	(0.94; (0.32–2.79) *p* = 0.91)	(0.46; (0.21–1.04) *p* = 0.06)
	Between $52,000 - $77,999	26 (15.9)	(0.87; (0.27–2.82) *p* = 0.81)	(0.24; (0.09–0.62) *p* = 0.00)
	$78,000 or more	52 (31.9)	(0.81; (0.31–2.11) *p* = 0.67)	(0.36; (0.17–0.73) *p* = 0.01)
Mutation	C-terminal	22 (9.4)	baseline comparison	
*n* = 234	Early truncating	16 (6.8)	(1.13; (0.24–5.24) *p* = 0.88)	(2.06; (0.57–7.47) *p* = 0.27)
	Large deletion	17 (7.3)	(0.94; (0.21–4.26) *p* = 0.94)	(4.93; (1.46–16.7) *p* = 0.01)
	p.Arg106Trp	11 (4.7)	(0.19; (0.02–1.42) *p* = 0.11)	(2.68; (0.66–10.92) *p* = 0.17)
	p.Arg133Cys	20 (8.6)	(0.68; (0.16–2.84) p = 0.6)	(2.43; (0.73–8.07) *p* = 0.15)
	p.Arg168*	28 (12.0)	(1.13; (0.31–4.16) *p* = 0.85)	(1.64; (0.53–5.08) *p* = 0.39)
	p.Arg255*	17 (7.3)	(0.27; (0.05–1.45) *p* = 0.13)	(2.09; (0.58–7.47) *p* = 0.26)
	p.Arg270*	18 (7.7)	(0.77; (0.18–3.38) *p* = 0.73)	(2.8; (0.83–9.38) *p* = 0.1)
	p.Arg294*	20 (8.6)	(0.68; (0.16–2.88) *p* = 0.6)	(1.97; (0.59–6.52) *p* = 0.27)
	p.Arg306Cys	14 (6.0)	(0.94; (0.2–4.42) p = 0.94)	(1.17; (0.31–4.5) *p* = 0.82)
	p.Thr158Met	25 (10.7)	(0.82; (0.21–3.21) *p* = 0.78)	(1.95; (0.61–6.2) *p* = 0.26)
	Other	26 (11.1)	(1.26; (0.39–4.73) p = 0.73)	(1.44; (0.46–4.47) *p* = 0.53)
Epilepsy	No	42 (17.6)	baseline comparison	
*n* = 237	Yes	196 (82.4)	(1.39; (0.59–3.26) *p* = 0.46)	(0.98; (0.51–1.91) *p* = 0.96)
Reflux	No	174 (73.1)	baseline comparison	
*n* = 238	Yes	64 (26.9)	(0.95; (0.47–1.92) *p* = 0.88)	(1.04; (0.59–1.83) *p* = 0.89)

^a^Refers to number of extracted teeth

When compared to those living in major cities the incidence of restorations was marginally higher for those living in inner regional Australia (Incidence Rate Ratio (IRR) 1.40; 95% CI 0.66–2.97), higher again for those living in outer regional Australia (IRR 1.64; 95% CI 0.59–4.52), and highest for those living in remote/very remote areas (IRR 1.86; 95% CI 0.44–7.82) (Table 3). However, the incidence of restorations for those in the lowest income bracket was similar to those in the other income brackets (Table 3).

When compared to those with a C-terminal deletion (see Table 3), the incidence of restorations was lower for those with a p.Arg106Trp mutation (IRR 0.19; 95% CI 0.02–1.42) and a p.Arg255* mutation (IRR 0.27; 95% CI 0.05–1.45). The incidence rate of restorations for cases with independent mobility was 8.3 per 100 py (95% CI 7.0–9.9) as shown in Table 2. Compared to those with no epilepsy, the incidence rate of restorations for those with epilepsy was higher at 7.0 per 100 py (IRR 1.39; 95% CI 0.59–3.26) (Table 3). Compared to those without reflux, the incidence rate of restorations for cases with reflux was slightly lower (Table 2) at 6.3 per 100 py (IRR 0.95; 95% CI 0.47–1.92).

### Incidence of extractions

The incidence rates of extractions by age group and covariates are presented in Table 2 and the incidence rate ratios in Table 3. The total incidence of extractions was 9.3 extractions per 100 py equating to just over nine teeth extracted if ten individuals were followed for ten years. Using the midpoint of age during the time period to which follow-up referred and excluding the first time point, the incidence of extractions was 10.0 per 100 py for the age group of 6 years or less; 12.6 per 100 py for 7–12 years of age, 11.9 per 100 py for 13–19 years of age and 23.8 per 100 py for 20 years and over (Table 2).

When compared to those living in major cities the incidence of extractions for those living in inner regional Australia (IRR 1.10; 95% CI 0.61–2.00), and those living in outer regional Australia (IRR 1.09; 95% CI 0.47–2.53) were fairly similar but slightly lower for those living in remote/very remote Australia (IRR 0.35; 95% CI 0.10–1.30).

The incidence of extractions by income was highest for those in the lowest income bracket of less than $31,200 p.a. with an incidence of 13.1 per 100 py (95% CI 10.9–15.7). Compared to the lowest income bracket the incidence of extractions was lower for those in the $31,200 - $51,999 income bracket (IRR 0.46; 95% CI 0.21–1.04), for those in the $52,000 - $77,999 bracket (IRR 0.24; 95% CI 0.09–0.62) and for those in the income bracket of $78,000 or more (IRR 0.36; 95% CI 0.17–0.73).

Compared with C-terminal deletions, the incidence of extractions for those with large deletions was higher at 20.3 per 100 py (Table 2) (IRR 4.93; 95% CI 1.46–16.7) (Table 3), and higher also at 13.9 per 100 py (Table 2) for those with p.Arg270* mutations (IRR 2.8; 95% CI 0.83–9.38). The incidence of extractions for those with epilepsy was similar (IRR 0.98; 95% CI 0.51–1.91) to those without epilepsy as it was for those with and without reflux (IRR 1.04; 95% CI 0.59–1.83).

### Dental attendances

The analysis of dental visit attendance by their respective covariates using data for the questionnaires received in 2009 and 2011 is shown in Table 4. Almost two thirds of the subjects, 61. 5% in 2009 and 62.8% in 2011, had a dental visit in the previous calendar year. There was little difference in attendance by geographical remoteness, family income, or mutation type. In 2009, a similar proportion of those with and without epilepsy were reported to have attended the dentist 61.1% and 63.6% respectively, but in 2011 a slightly higher proportion of those with epilepsy (65.8%) attended compared to those without epilepsy (50.0%) (Table 4). Analysis of the 2009 data also showed that a higher proportion of those for whom gastric reflux was reported had attended a dentist (74.5%) compared to those for whom reflux was not reported (56.3%) although there was little difference between the groups in 2011 (Table 4).

**Table 4:** Dental visits by covariates

Wave		Dental visit	2009^a^							2011^a^						
			Yes		No		Total		*P* value^b^	Yes		No		Total		*P* value^b^
ARIA+ category	Major cities		65	(66.3)	33	(33.7)	98	(100.0)	*p* = 0.13	74	(67.3)	36	(32.7)	110	(100.0)	*p* = 0.15
	Inner regional		24	(57.1)	18	(42.9)	42	(100.0)		26	(63.4)	15	(36.6)	41	(100.0)	
	Outer regional		7	(38.9)	11	(61.1)	18	(100.0)		8	(42.1)	11	(57.9)	19	(100.0)	
	Remote/very remote		6	(75.0)	2	(25.0)	8	(100.0)		5	(50.0)	5	(50.0)	10	(100.0)	
	Total		102	(61.4)	64	(38.6)	166	(100.0)		113	(62.8)	67	(37.2)	180	(100.0)	
Family income	Less than $31,200		19	(55.9)	15	(44.1)	34	(100.0)	*p* = 1.00	18	(51.4)	17	(48.6)	35	(100.0)	*p* = 0.13
	Between $31,200 - $51,999		12	(54.6)	10	(45.4)	22	(100.0)		17	(73.9)	6	(26.1)	23	(100.0)	
	Between $52,000 - $77,999		10	(58.8)	7	(41.2)	17	(100.0)		11	(47.8)	12	(52.2)	23	(100.0)	
	$78,000 or more		21	(56.8)	16	(43.2)	37	(100.0)		29	(69.0)	13	(31.0)	42	(100.0)	
	Total		62	(56.4)	48	(43.6)	110	(100.0)		76	(61.0)	48	(39.0)	123	(100.0)	
Epilepsy	No		14	(63.6)	8	(36.4)	22	(100.0)	*p* = 1.00	17	(50.0)	17	(50.0)	34	(100.0)	*p* = 0.12
	Yes		88	(61.1)	56	(38.9)	144	(100.0)		96	(65.8)	50	(34.2)	146	(100.0)	
	Total		102	(61.4)	64	(38.6)	166	(100.0)		113	(62.8)	67	(37.2)	180	(100.0)	
Reflux	No		67	(56.3)	52	(43.7)	119	(100.0)	*p* = 0.03	78	(60.5)	51	(39.5)	129	(100.0)	*p* = 0.39
	Yes		35	(74.5)	12	(25.5)	47	(100.0)		35	(68.6)	16	(31.4)	51	(100.0)	
	Total		102	(61.4)	64	(38.6)	166	(100.0)		113	(62.8)	67	(37.2)	180	(100.0)	
Mutation	C-terminal		12	(66.7)	6	(33.3)	18	(100.0)	*p* = 0.95	11	(68.8)	5	(31.2)	16	(100.0)	p = 0.88
	Early truncating		8	(72.7)	3	(27.3)	11	(100.0)		8	(66.7)	4	(33.3)	12	(100.0)	
	Large deletion		8	(66.7)	4	(33.3)	12	(100.0)		8	(57.1)	6	(42.9)	14	(100.0)	
	p.Arg106Trp		3	(50.0)	3	(50.0)	6	(100.0)		4	(44.4)	5	(55.6)	9	(100.0)	
	p.Arg133Cys		9	(52.9)	8	(47.1)	17	(100.0)		10	(52.6)	9	(47.4)	19	(100.0)	
	p.Arg168*		8	(61.5)	5	(38.5)	13	(100.0)		13	(61.9)	8	(38.1)	21	(100.0)	
	p.Arg255*		10	(66.7)	5	(33.3)	15	(100.0)		7	(53.8)	6	(46.2)	13	(100.0)	
	p.Arg270*		9	(60.0)	6	(40.0)	15	(100.0)		9	(69.2)	4	(30.8)	13	(100.0)	
	p.Arg294*		7	(46.7)	8	(53.3)	15	(100.0)		10	(76.9)	3	(23.1)	13	(100.0)	
	p.Arg306Cys		4	(50.0)	4	(50.0)	8	(100.0)		5	(50.0)	5	(50.0)	10	(100.0)	
	p.Thr158Met		13	(72.2)	5	(27.8)	18	(100.0)		14	(73.7)	5	(26.3)	19	(100.0)	
	Other		10	(58.8)	7	(41.2)	17	(100.0)		11	(61.1)	7	(38.9)	18	(100.0)	
	Total		102	(61.2)	64	(38.8)	165	(100.0)		110	(62.2)	67	(37.6)	177	(100.0)	

^a^Figures quoted as *n* (%) ^b^Fisher’s exact test of independence

In a separate longitudinal analysis it was found that cases with mild bruxism (RR 1.05; 95% CI 0.89–1.30) and severe bruxism (RR 1.05; 95% CI 0.84–1.32) were 5% more likely to have attended the dentist in the reported time period, with adjustment for mutation type.

### Receipt of dental treatment under GA

In 2009, dental treatment under GA was reported for 24 out of a total 77 persons, and 24 out of a total 79 cases in 2011 with a mode value of one dental extraction (Table 5). Descriptive data on the number of dental extractions and restorations performed under GA in the 2009 and 2011 time periods are shown in Table 6. The median number of restorations under GA for the 2009 time period was two restorations, and the mode for the number of restorations under GA for the 2011 time period was two restorations. The receipt of dental treatment under GA for the 2009 and 2011 time periods by their respective covariates is shown in Table 5.

**Table 5:** Dental receipt of general anaesthesia by covariates

Wave		Dental treatment GA	2009^a^							2011^a^						
			Yes		No		Total		*P* value^b^	Yes		No		Total		*P* value^b^
ARIA+ category	Major cities		13	(26.5)	36	(73.5)	49	(100.0)	*p* = 0.52	12	(24.0)	38	(76.0)	50	(100.0)	*p* = 0.14
	Inner regional		8	(44.4)	10	(55.6)	18	(100.0)		10	(50.0)	10	(50.0)	20	(100.0)	
	Outer regional		1	(20.0)	4	(80.0)	5	(100.0)		1	(16.7)	5	(83.3)	6	(100.0)	
	Remote/very remote		2	(40.0)	3	(60.0)	5	(100.0)		1	(33.3)	2	(66.7)	3	(100.0)	
	Total		24	(31.2)	53	(68.8)	77	(100.0)		24	(30.4)	55	(69.6)	79	(100.0)	
Family income	Less than $31,200		2	(13.3)	13	(86.7)	15	(100.0)	*p* = 0.67	5	(50.0)	6	(50.0)	12	(100.0)	*p* = 0.21
	Between $31,200 - $51,999		3	(33.3)	6	(66.7)	9	(100.0)		1	(10.0)	9	(90.0)	10	(100.0)	
	Between $52,000 - $77,999		1	(14.3)	6	(85.7)	7	(100.0)		3	(37.5)	5	(62.5)	8	(100.0)	
	$78,000 or more		4	(26.7)	11	(73.3)	15	(100.0)		6	(25.0)	18	(75.0)	24	(100.0)	
	Total		10	(21.7)	36	(78.3)	46	(100.0)		16	(29.6)	38	(70.4)	54	(100.0)	
Epilepsy	No		3	(37.5)	5	(62.5)	8	(100.0)	*p* = 0.70	2	(18.2)	9	(81.8)	11	(100.0)	*p* = 0.49
	Yes		21	(30.4)	48	(69.6)	69	(100.0)		22	(32.4)	46	(67.6)	68	(100.0)	
	Total		24	(31.2)	53	(68.8)	77	(100.0)		24	(30.4)	55	(69.6)	79	(100.0)	
Reflux	No		15	(31.9)	32	(68.1)	47	(100.0)	*p* = 1.00	17	(30.4)	39	(69.6)	56	(100.0)	*p* = 1.00
	Yes		9	(30.0)	21	(70.0)	30	(100.0)		7	(30.4)	16	(69.6)	23	(100.0)	
	Total		24	(31.2)	53	(68.8)	77	(100.0)		24	(30.4)	55	(69.6)	79	(100.0)	
Mutation	C-terminal		2	(18.2)	9	(81.8)	11	(100.0)	*p* = 0.30	1	(11.1)	8	(88.9)	9	(100.0)	*p* = 0.16
	Early truncating		1	(16.7)	5	(83.3)	6	(100.0)		4	(57.1)	3	(42.9)	7	(100.0)	
	Large deletion		1	(12.5)	7	(87.5)	8	(100.0)		2	(33.3)	4	(66.7)	6	(100.0)	
	p.Arg106Trp		2	(66.7)	1	(33.3)	3	(100.0)		2	(66.7)	1	(33.3)	3	(100.0)	
	p.Arg133Cys		1	(50.0)	1	(50.0)	2	(100.0)		0	(0.0)	8	(100.0)	8	(100.0)	
	p.Arg168*		3	(60.0)	2	(40.0)	5	(100.0)		2	(25.0)	6	(75.0)	8	(100.0)	
	p.Arg255*		4	(50.0)	4	(50.0)	8	(100.0)		3	(60.0)	2	(40.0)	5	(100.0)	
	p.Arg270*		2	(28.6)	5	(71.3)	7	(100.0)		1	(25.0)	3	(75.0)	4	(100.0)	
	p.Arg294*		3	(60.0)	2	(40.0)	5	(100.0)		2	(40.0)	3	(60.0)	5	(100.0)	
	p.Arg306Cys		1	(33.3)	2	(66.7)	3	(100.0)		2	(50.0)	2	(50.0)	4	(100.0)	
	p.Thr158Met		3	(37.5)	5	(62.5)	8	(100.0)		2	(18.2)	9	(81.8)	11	(100.0)	
	Other		1	(10.0)	9	(90.0)	10	(100.0)		3	(50.0)	3	(50.0)	6	(100.0)	
	Total		24	(31.6)	52	(68.4)	76	(100.0)		24	(31.6)	52	(68.4)	76	(100.0)	

^a^Figures quoted as *n* (%) ^b^Fisher’s exact test of independence

**Table 6:** Dental extractions and restorations received under general anaesthesia

Year	Restoration				Extraction			
	*n*	Frequency	%	% (Cumulative)	*n*	Frequency	%	% (Cumulative)
2009	0	1	8.33	8.33	0	1	6.25	6.25
*n* = 102	1	3	25	33.33	1	5	31.25	37.5
	2	3	25	58.33	2	3	18.75	56.25
	3	1	8.33	66.67	4	5	31.25	87.5
	4	3	25	91.67	11	1	6.25	93.75
	7	1	8.33	100	12	1	6.25	100
	Total	12	100		Total	16	100	
2011	1	1	12.5	12.5	0	1	10	10
*n* = 113	2	4	50	62.5	1	4	40	50
	4	1	12.5	75	3	1	10	60
	6	1	12.5	87.5	4	2	20	80
	18	1	12.5	100	5	1	10	90
					28	1	10	100
	Total	8	100		Total	10	100	

Dental treatment under GA was slightly higher in those living in inner regional Australia in both the 2009 and 2011 data (8/18 and 10/20 respectively) compared to those living in the major cities of Australia (13/49 and 12/50 respectively). The proportion of those living in outer regional Australia with dental treatment reported under GA were 1/5 and 1/6 respectively for the years 2009 and 2011, lower compared to those living in the major cities of Australia. Dental treatment under GA was also reported slightly more frequently (2/5 and 1/3 respectively) for those living in remote/very remote Australia as compared to those in major cities of Australia (Table 5).

Slightly less than one third of those subjects with epilepsy (21/69 in 2009 and 22/68 in 2011) were reported to have received dental treatment under GA, as compared to 3/8 of those without epilepsy in 2009 and 2/11 in 2011 (Table 5). Examination of both the 2009 and 2011 data revealed unremarkable differences between the receipt of GA between those with and without gastric reflux (Table 5).

### Other dental treatments and problems

Gingival bleeding was reported for 70/116 (60.7%) cases in 2009 and 124/210 (59.1%) in 2011. Other dental problems included dental trauma from falls, bruxism or malocclusion.

A history of dental trauma was reported for 22 cases, with a fall as the mechanism of injury in 12 and trauma in association with a seizure in four. One individual was hit while at school. Reported treatment for trauma included implantation of avulsed teeth (*n* = 2), restoration (*n* = 5), extraction (*n* = 2), placement of a dental implant (*n* = 1), root canal treatment (*n* = 1) and no treatment (*n* = 5) with four of these cases involving avulsed teeth which were not reimplanted. Bruxism was reported as a dental problem by 33 (13.6%) respondents, with associated dental treatment being extraction (*n* = 3), sealants (*n* = 4), use of a splint (*n* = 4), stainless steel crowns (*n* = 4), veneers (*n* = 1), and botox (*n* = 1). Tooth wear was also stated as a dental problem in ten cases, with reported treatment as being extraction (*n* = 2), and placement of a sealant (*n* = 1). Nineteen respondents (7.9%) reported malocclusion as a dental problem, with crowding as the most common issue (*n* = 13) followed by open-bite (*n* = 2) and protrusive teeth (*n* = 2). There was one report of extraction being performed to relieve crowding of the teeth.

### Relationship between bruxism and age

**Fig. 1:**
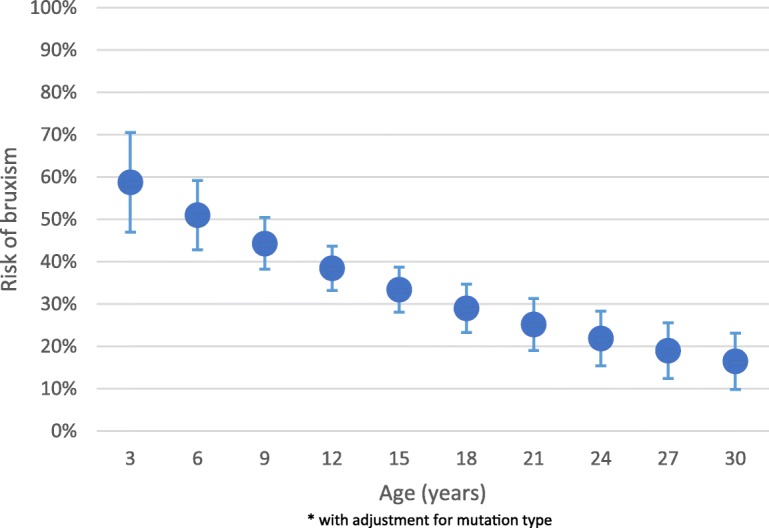
Predictive risk of frequent bruxism by age

Longitudinal analysis showed a 5% decrease in risk for frequent bruxism for every one year increase in age, after adjusting for mutation type (RR 0.95; 95% CI 0.94–0.97). This translates to a predictive risk of bruxism (Fig. 1) of 58.74% at three years of age, 38.46% at twelve years of age and 16.49% at 30 years of age, with adjustment for mutation type.

## Discussion

### Principal findings

This study found that both restoration and extraction of teeth were occurred only rarely in this population at a rate of less than one in ten years, although extractions were more common than restorations. Extractions occurred a little more frequently in those whose families had lower incomes, and in those subjects with a more severe genotype. The predictive risk of bruxism decreased with age but was reported to be a dental problem by 33 (13.6%) respondents. Those for whom bruxism had been reported were a little more likely to have attended the dentist in the reported time period. Additional to bruxism, other dental problems reported included gingival bleeding in more than half of the subjects; and dental trauma from falls, bruxism or malocclusion.

### Strengths and limitations of the study

The foundation of our dataset was the population and longitudinal ARSD that has evolved to include aspects of dental health. It is the only dataset of its type in the world as it is the only existing population-based register of individuals with RTT, and has ongoing ascertainment through the Australian Paediatric Surveillance Unit. Additionally, the ARSD contains information on specific genetic mutations and phenotypic characteristics of the individuals. This is gathered from both clinician-based and caregiver-based questionnaires, with longitudinal data collected every two to four years. These features make it the most comprehensive database of its type in the world. Whilst only the 2009 and 2011 family questionnaires could be analysed for trends in dental visits and for the receipt of dental treatment under GA, nonetheless ours is the first study to investigate dental issues over time in RTT. We acknowledge some limitations. Firstly, there were missing data in relation to family income level and it is possible that there may have been an underascertainment of those in the highest income group in our income level analyses. Secondly, the highest ARIA+ score was used as the individual’s allocated ARIA+ score for analysis, and so the analyses may overestimate the effect of geographic remoteness as a co-variate. Thirdly, using the mid-point of the time periods reported as the allocated age for the incidence calculations had varied implications for analysis and comparison of the data. The method used to determine age meant that incidence figures for restorations and extractions by age group were approximations. Furthermore, it is impossible to compare these figures with existing dmft/DMFT (decayed, missing, filled teeth in the primary dentition and permanent dentition respectively) components published in the literature of a control or “normal” population, as the dmft/DMFT is based on a precise measure of an individual’s dental charting at one point in time. Finally, it was unclear whether or not formal diagnoses of gastro oesophageal disease (GORD) were made, and this would provide useful information. Because of the limitations in power, mainly associated with the rarity of the dental outcomes, many results from the analyses did not approach statistical significance.

### Strengths and weaknesses in relation to other studies

This is the first study to have examined the rate of restorations and extractions in a population-based RTT cohort. It has demonstrated that these procedures are both occurring infrequently at a rate of less than one per ten years, while extractions were slightly commoner than restorations. These figures are not directly comparable with decayed, missing, filled teeth indices reported in the general population as these are gathered prospectively on examination. One Spanish study did compare selected oral health outcomes of 41 girls with RTT with a control sample, however key indices were not available for primary teeth and it was not a population-based cohort and the results were not comparable with ours [35]. In our RTT population, restorations occurred a little more frequently and extractions a little less frequently with increasing geographical remoteness. This is in contrast to the broader dental literature, which suggests that oral health is poorer in rural areas compared to major city areas in Australia [46]. Whilst the incidence rates of restorations did not vary by family income, we found a slightly higher rate of extractions in those whose families had lower incomes. Perhaps more extractions were being performed at the expense of restorations in more disadvantaged families because of less resources for preventive care. Similar relationships between socioeconomic status (SES) and oral health have been shown in the wider dental literature [47, 48].

There did not appear to be obvious patterns in dental attendance by family income levels. In contrast, Australian statistics report a significant difference in dental attendance in the lowest two household income categories (less than $30,000, and $30,000 to less than $60,000; 57.1% and 56.8%, respectively) as compared to the highest two income categories ($90,000 to $140,000, and $140,000 plus; 66.9% and 67.9%, respectively) in the previous 12 months in 2013 [49]. One possible explanation may be that children with RTT qualify for treatment in specialised children’s hospitals in some states in Australia, thereby providing a service that may not otherwise be afforded by low income families who cannot afford to seek dental care in the private sector. Unfortunately, some states in Australia do not provide a transition service for these girls from childhood through adolescence and into adulthood, or access to special needs dentists, and so this may be a confounding factor. The patterns of dental attendance by geographical remoteness appeared similar in the 2009 and 2011 time periods. Interestingly, dental attendance in very remote and remote areas exceeded dental attendance in outer regional areas while treatment under GA was reported in slightly more cases living in inner regional areas compared to major cities of Australia. These findings may relate to the availability of transport schemes to reduce the impacts of living in remote areas. It is probable that these children could be brought into major treatment centres sometimes by air for multi-discipline appointments, which could include dental visits. This contrasts with national statistics that indicate that the proportion of people in Australia aged 15 years and over attending the dentist in the previous 12 months in 2013 was higher in those living in major cities as compared to those in inner regional or outer regional areas [49].

This is also the first study to have examined the longitudinal trajectory of bruxism and we found that it declined with increasing age. Both nocturnal and diurnal bruxism have been reported in larger studies. For example one study (*n* = 126) reported diurnal teeth grinding in 60% (*n* = 69), nocturnal teeth grinding in 27% (*n* = 28), and teeth clenching in 54% of individuals [36]. A study comparing the behavioural phenotype of girls with RTT (*n* = 143) to those with severe mental retardation (SMR) (*n* = 85) using the Rett Syndrome Behaviour Questionnaire (RSBQ) reported teeth grinding in 86.0% of those with RTT and 44.7% in the SMR group [40]. One comparative study identified a higher degree of tooth wear in girls with RTT (*n* = 41) when compared to an age-matched control population (*n* = 82) in Spain, as measured by the O’Brien erosion index [35]. However, it was not clear as to whether the tooth wear reported was due to parafunctional habits, erosion or otherwise.

### Meaning of the study: Possible mechanisms and implications for clinicians or policymakers

There are no incidence figures in the literature that would allow comparison of figures from the present study with a normal population group because oral health outcomes are usually recorded as a prospective record of decayed, missing and filled teeth (DMFT/dmft). However, our data would suggest that dental procedures are comparatively less common in individuals with RTT than in the general population. Whether this is because of better oral health or because of poor access to services is unclear. Since these children and adults are generally not able to self-feed, they would have relatively less access to high sugar foods, but, conversely, teeth-cleaning is much more challenging to perform efficiently and is carried out by the caregiver. The absence of gingival bleeding is a positive indicator of periodontal stability [50] and we would estimate from our data that approximately one third of cases could be assumed to have good gingival health.

General clinical severity also appeared to play a role in dental health. The incidence of extractions was higher for both large deletions (*n* = 50 in 17 females) and p.Arg270* (*n* = 50 in 18 females) mutations as compared to C-terminal mutations. It is noteworthy that the C-terminal deletion mutation is associated with a milder phenotype while large deletions, p.Arg270* and p.Arg255* mutations have a more severe phenotype [31, 32, 51, 52] including poorer hand use, ambulation and language skills. As observed with those with more severe mutations, this could be reflective of a shift in management from treatment to palliation in girls who are more incapacitated. Reported dental attendance was similar between those with and without epilepsy, although slightly higher for those in whom reflux was reported. This could possibly reflect caregiver’s concerns over the dental effects of reflux. Although gastric reflux is known to be related to dental erosion, there is currently no available literature on the dental effects of reflux in subjects with RTT. Similarly, the rate of restorations and extractions did not differ greatly for those with or without epilepsy or gastric reflux. One may suppose that those with epilepsy may be more prone to trauma to anterior teeth from seizures, but those without epilepsy, who are still able to walk, may still be prone to anterior tooth trauma from falls because of ataxia.

The study’s findings on the longitudinal trajectory of bruxism found that it declined with increasing age. This is important information when counselling families. Those with bruxism were a little more likely to have attended the dentist. It is possible that this also may be a reflection of the caregivers’ attitudes and concerns over the effect of bruxism on the dentition and the fact that bruxism is perceived as a dental problem.

### Unanswered questions and future research

It is interesting to reflect on a possible influence of *MECP2* on oral health. Bone mass and density are typically poor in females with RTT [26, 53] and animal studies are suggesting a role for *MECP2* in bone development [54–56]. It is also established that Vitamin D levels affect bone health [57] and vitamin D deficiency was reported in 20% of RTT subjects in one study (*n* = 154) [58]. Current research suggests that bone health and oral health are related and that osteoporosis seems to independently affect alveolar bone levels [59]. The authors speculate that genotypes associated with a more severe phenotype such as the large deletion may be associated with poorer oral as well as bone health but further investigation would be required.

Our data do provide some important insights into the dental health of the RTT population, although many of the results did not reach statistical significance likely due to the rarity of the dental outcomes. The rate of caries may be low because of the beneficial effects of a healthy diet. Conversely, the low rate of dental procedures may be due to the lack of access to services. Further research could replicate investigations from this study in a larger model with a powered sample, and this could be achieved through the use of an existing international database.
